# Analytical Model for Early Design Stage of Cable-Stayed Suspension Bridges Based on Hellinger–Reissner Variational Method

**DOI:** 10.3390/ma15144863

**Published:** 2022-07-12

**Authors:** Qian Feng, Peng Wei, Junbin Lou, Daiwei Wang, Jinbiao Cai, Rongqiao Xu

**Affiliations:** 1 Center for Balance Architecture, Zhejiang University, Hangzhou 310007, China; fengqian@zju.edu.cn (Q.F.); 21812082@zju.edu.cn (P.W.); 22112279@zju.edu.cn (J.L.); caijb@zju.edu.cn (J.C.); 2 Department of Civil Engineering, Zhejiang University, Hangzhou 310058, China; 3 China United Engineering Corporation Limited, Hangzhou 310051, China; wangdw@chinacuc.com

**Keywords:** cable-stayed suspension bridge, design stage, Hellinger–Reissner variational principle, deformation, force

## Abstract

The cable-stayed suspension bridge is one type of bridge that has been increasingly applied to bridge engineering, especially in cross-sea projects. However, the complex combined system of this type of bridge makes it quite difficult for researchers to make a quick decision of the parameter values during the design stage. The Hellinger–Reissner method is applied here to analyze the deformation and force of the structural members in the bridge. The advantage of this method is that the solving of deformation and force is independent of each other, which would enhance the accuracy of the final results. Different load conditions are also considered in the analysis. The results from the present method are compared with test results and finite element analysis, and show good agreements. It implies that the Hellinger–Reissner is a comparatively more efficient method to help designers choose the key parameters for cable-stayed suspension bridges.

## 1. Introduction

**Figure 1:**
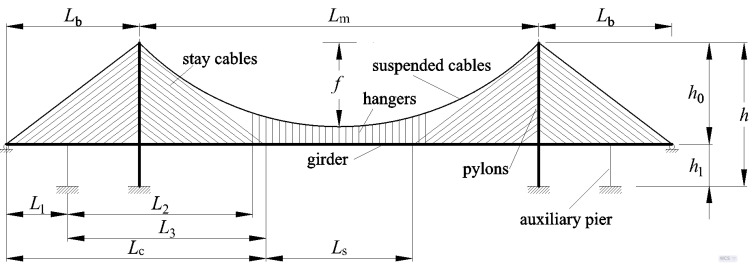
Structural members in the cable-stayed suspension bridge.

The cable-stayed suspension bridge is a combination of the cable-stayed system and the suspension system [1]. The structural members in the cable-stayed suspension bridge are introduced in Figure 1. Compared with suspension bridges, the cable-stayed suspension bridges have higher structural rigidity [1,2]. Compared with cable-stayed bridges, the cable-stayed suspension bridge provides better stability because of the lower compressive force caused by bridge decks. Furthermore, it has a lower cost than a suspension bridge with the same bridge span. Hence, the cable-stayed suspension bridge is an excellent solution for long-span bridges across the sea for structural, economic, and practical reasons [1,2,3].

There has been a large amount of research work on cable-stayed bridges and suspension bridges, including analyses of static, dynamic, linear, and nonlinear behaviors [4,5,6,7,8,9,10,11,12,13,14,15]. However, very few analyses have been conducted on the combined cable-stayed suspension bridges. Gimsing [1] has introduced the history and development of this type of bridge and provided detailed description of the two categories of this combined system: Roebling’s structural system and Dichinger’s structural system. Sun et al. [2] presented a four-step approach for determining the finished dead load state of a cable-stayed suspension bridge during the design stage by combining a finite element analysis with an iterative numerical method. Konstantakopoulos and Michaltsos [16] presented a mathematical model to analyze the nonlinear behavior of this type of bridge. Experimental investigation has also been applied to the study of the stability of the cable-stayed suspension bridge with orthotropic steel boxes [17]. Tests using models built in the laboratories were also conducted for optimizing the results of analyses [18,19].

The cable-stayed suspension bridges are still not as commonly constructed as cable-stayed bridges or suspension bridges. One of the major reasons is that there are interactions within the cable-stayed system and the suspension system in the design stage [1,16,20]. Analyses of complex combined cable systems are difficult because they not only require a large amount of work, but also could lead to significant errors. The design stage normally could be divided into several stages. First, in the early design stage, the bridge types along with corresponding material properties should be decided. The deformation and forces should be calculated to see if the chosen material and dimensional properties satisfy the early design stage requirements. Some approximate solutions such as the Ritz method have been presented to help in choosing parameters for the early design stage and solving the problems of engineering projects [21,22,23,24,25,26]. This method is highly efficient in obtaining the deformation of the members. In the Ritz method, the deformation hypothesis is the only hypothesis at the beginning of the computing process. Therefore, the disadvantage of the Ritz method is that the determination of the forces relies on the deformation solutions. The indirect computation of forces would lower the accuracy significantly. It is extremely important to obtain the key parameters of bridge members efficiently and correctly. Once the bridge type, the corresponding material, and dimensional properties are decided, the detailed structural analysis would be carried out, including more complicated loading combinations, loading types, dynamic behaviors, shear impacts, torsion effects, etc.

The Hellinger–Reissner variational principle is introduced here for the design of the cable-stayed suspension bridge. There are several parts included in the formulations of the Hellinger–Reissner functional [27,28]. The steps of this method mainly include raising the trial functions of the deformations and inner forces, applying the trial functions to the expression of the Hellinger–Reissner method, bringing in the deformation compatibility conditions, solving the equations, and obtaining the expressions for the deformation and inner forces. This method has been used by researchers to solve some engineering problems such as shells, beams, or new elements by incorporating with finite element analysis [29,30,31,32,33,34,35,36]. This is the first time that the Hellinger–Reissner method is applied for the analysis of the cable-stayed suspension bridge. The advantage of this method is that the expressions of the deformations and forces are independent of each other, instead of obtaining inner forces from the deformation. Therefore, it enhances the accuracy of forces to a large degree. This study also investigates the deformation and forces under different combinations of loads and sag-to-span ratios. The results show that the present method would help engineers make quick decisions on choosing the key parameters of the cable-stayed suspension bridge in the design stage. 

## 2. Mechanical Model for Early Design Stage

**Figure 2:**
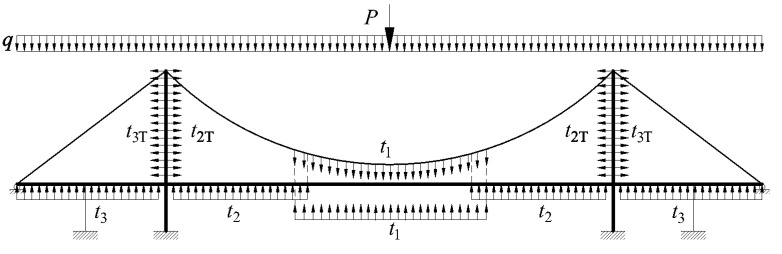
Simplified combined cable-stayed suspension system.

The cable-stayed suspension bridge is a composite of many complex structural members. The following assumptions are suggested for the analysis in the conceptual design stage:

(1) A symmetrical three-span cable-stayed suspension bridge with two pylons is taken as an example to analyze this type of bridge, as shown in Figure 1. The girder of the suspension and girder of the cable-stayed part are linked continuously. The fully floating deck system is considered in the analysis because of the presence of the auxiliary pier on the side span. This means that the pylon and girder in this case are not connected with any linkage vertically and longitudinally, but are still coupled in the lateral direction. Otherwise, without any auxiliary pier, the semi-floating deck system is applied here to simulate the additional linkage connecting the girder and pylon in the vertical direction.
(2) The hangers and stay cables are distributed uniformly along the girder of the suspension and girder of the cable-stayed part, respectively. According to the membrane analogy, the force effects of the hangers and cables could be equivalent to those of a membrane between the catenary and girder. The rationality of this step is also supported by the results of a previous study [16]. As shown in Figure 2, the forces in the hangers can be replaced by a uniformly distributed load *t*_1_. Meanwhile, for the stay cables connecting the pylon and girder in the main span, the forces in the stay cables are equivalent to the uniformly distributed load *t*_2_ acting on the girder and *t*_2T_ acting on the pylon. Similarly, in the side span, the forces in the stay cables are changed into a uniformly distributed load *t*_3_ acting on the girder and *t*_3T_ acting on the pylon.
(3) The live load caused by traffic and walking people could be replaced by a distributed load and concentrated load for static analysis. Meanwhile, the dead load of the bridge could also be equivalent to a uniformly distributed load. According to the superimposition principle, the load on the bridge can be assumed to be a combination of the distributed load *q* and concentrated load *P*, as shown in Figure 2. The different combinations of the distributed load *q* and concentrated load *P* are considered in the following calculation.
(4) The axial force and deformation of either the main beam or pylon are neglected. Hence, the corresponding terms of the strain energy are also neglected. The effect of the shear force of the main beam and pylon is also not considered here. The strain energy of the auxiliary pier is neglected.

## 3. Analysis

With the mechanic model of the cable-stayed suspension system, the Hellinger–Reissner method was adopted to analyze the cable-stayed suspension bridge. The steps for data processing are as follows:

(1) Trial functions of force and deformation should be proposed first with the coefficients to be solved. In this stage, the engineer is required to have the basic knowledge about how the structural members would deform with the load.
(2) The trial functions are then applied to the Hellinger–Reissner principle to obtain the expression of the functional.
(3) Partial differential equations for individual coefficients are obtained.
(4) The deformation compatibility conditions are introduced to obtain the following equations.
(5) By combining the equations from Step 3 and Step 4, the expressions of the coefficients are obtained by solving the equation sets.
(6) By employing the expressions of the coefficients in the trial functions of force and deformation, the force and deformation expressions are obtained.

### 3.1. Girder

#### 3.1.1. Hypotheses of Trial Functions

(1) Force functions:

During the early design stage, engineers focus more on the forces and deformations in several vital cross-sectional areas. Higher-order functions would make the computing results more accurate but would make the computing procedures more complicated. Hence, the trial function of the bending moment of the cable-stayed part *M*_1_ uses the linear function hypotheses. Function *M*_1_ is divided into two parts, *M*_11_ and *M*_12_, to fit the force distribution with the support from the pylon and auxiliary pier. The hypothesis of the bending moment trial function of the cable-stayed part from 0 to *L*_1_ is named *M*_11_. The second part, *L*_1_–*L*_c_, is named *M*_12_. 

The suspension part uses a quadratic equation as the hypothesis of the bending moment function, *M*_2_.

(2) Deformation function:

To simplify the computing procedures, deformation hypotheses are provided under the local coordinate system. The deformation function of the cable-stayed part *w*_1_ and that of the suspension part *w*_2_ both used quartic functions. Meanwhile, the deformation function *w*_1_ is a composite of two piecewise functions *w*_11_ and *w*_12_, corresponding to the force functions *M*_11_ and *M*_12_, respectively. The piecewise function *w*_12_ adopts the same function formation as *w*_11_ based on the coordinate translation.

#### 3.1.2. Derivation of Equations

The hypotheses of the trial functions for the deformation and force of the girder are as follows:

(1) For the cable-stayed part from 0 to *L*_1_, as shown in Figure 1, which is also from 0 to *L*_1_ in the local coordinate system, the deformation and force assumptions are described as follows:

(1) $$w_{11}=b_{1}x(x^{3}-L_{1}{}^{3})+b_{2}x(x^{2}-L_{1}{}^{2})\;$$

(2) $$M_{11}=e_{1}x+e_{2}\;$$

where *b*_1_, *b*_2_, *e*_1_, and *e*_2_ are the coefficients to be solved.

(2) For the cable-stayed part from *L*_1_ to *L*_c_ in the global coordinate system, as shown in Figure 1, the location in the local coordinate system ranges from 0 to *L*_3_. The deformation and force expressions are as follows:

(3) $$w_{12}=b_{1}(x+L_{1})[{\left(x+L_{1}\right)}^{3}-L_{1}{}^{3}]+b_{2}(x+L_{1})[{\left(x+L_{1}\right)}^{2}-L_{1}{}^{2}]\;$$

(4) $$M_{12}=e_{3}x+e_{4}\;$$

where *e*_3_ and *e*_4_ are the coefficients to be solved.

(3) For the suspension part from *L*_c_ to (*L*_c_+*L*_s_) in the global coordinate system, as shown in Figure 1, while the location is from 0 to *L*_s_ in the local coordinate system, the deformation and force expressions are as follows:

(5) $$w_{2}=a_{1}x^{2}{\left(x-L_{s}\right)}^{2}+a_{2}x(x^{3}-2L_{s}x^{2}+L_{s}{}^{3})+a_{3}\;$$

(6) $$M_{2}=e_{5}{\left(x-\frac{L_{s}}{2}\right)}^{2}+e_{6}\;$$

where *L*_3_ equals (*L*_c_−*L*_1_), which is the distance from the auxiliary pier or the support of the pylon to the first hanger that does not cross the stay cables, and *a*_1_–*a*_3_ and *e*_5_–*e*_6_ are the coefficients to be solved. 

Meanwhile, the continuous conditions should be satisfied because of the piecewise functions adopted in the deformation functions. The continuous conditions are described as *w*_2_|*_x_*_=0_ = *w*_12_|*_x_*_=*L*__3_, (*dw*_2_/*dx*)|*_x_*_=0_ = (d*w*_12_/*dx*)|*_x_*_=*L*__3_, and (*d*^2^*w*_2_/*dx*^2^)|*_x_*_=0_ = (d^2^*w*_12_/*dx*^2^)|*_x_*_=*L*__3_.

Which gives the following solution of *a*_1_, *a*_2_, and *a*_3_:

(7) $$a_{1}=\frac{6b_{1}L_{c}{}^{2}+3b_{2}L_{c}}{L_{s}{}^{2}}\;$$

(8) $$a_{2}=\frac{b_{1}(4L_{c}{}^{3}-L_{1}{}^{3})+b_{2}(3L_{c}{}^{2}-L_{1}{}^{2})}{L_{s}{}^{3}}\;$$

(9) $$a_{3}=b_{1}(L_{c}{}^{4}-L_{1}{}^{3})+b_{2}(L_{c}{}^{3}-L_{1}{}^{2})$$

Hence, only two coefficients, *b*_1_ and *b*_2_, are to be solved. 

The piecewise functions of the force should also satisfy the continuous conditions *M*_11_|*_x_*_= *L*__1_ = *M*_12_|*_x_*_=*0*_ and *M*_12_|*_x_*_= *L*__3_ = *M*_2_|*_x_*_=*0*_.

The coefficients *e*_4_ and *e*_6_ are solved and eliminated by this step, which gives the following expressions for *e*_4_ and *e*_6_: *e*_4_ = *e*_1_*L*_1_ + *e*_2_, *e*_4_ = *e*_1_*L*_1_ + *e*_2_ + *e*_3_*L*_3_ – (*e*_5_*L_s_*^2^)/4.

In summary, the expression of the girder part in the Hellinger–Reissner principle is as follows:

(10) $$\begin{array}{l} \pi_{\mathrm{HRb}}=2\int_{0}^{L_{1}}\left(-M_{11}\right)\left(\frac{d^{2}w_{11}}{dx^{2}}\right)dx-\int_{0}^{L_{1}}\frac{M_{11}{}^{2}}{E_{b1}I_{b1}}dx\;+2\int_{0}^{L_{3}}\left(-M_{12}\right)\left(\frac{d^{2}w_{12}}{dx^{2}}\right)dx-\int_{0}^{L_{3}}\frac{M_{12}{}^{2}}{E_{b1}I_{b1}}dx\\ \;\;\;\;\;\;\;\;\;+\int_{0}^{L_{s}}\left(-M_{2}\right)\left(\frac{d^{2}w_{2}}{dx^{2}}\right)dx-\frac{1}{2}\int_{0}^{L_{s}}\frac{M_{2}{}^{2}}{E_{b2}I_{b2}}dx \end{array}$$

The simplified formation of the equation could be expressed as:

(11) $$\pi_{\mathrm{HRb}}=\xi_{b}{}^{T}K_{b}\xi_{b}\;$$

where $\xi_{b}={\left[\begin{array}{cccccc} e_{1} & e_{2} & e_{3} & e_{5} & b_{1} & b_{2} \end{array}\right]}^{T}$, $K_{b}=\left[\begin{array}{cccccc} 2R_{1} & R_{5} & R_{6} & R_{7} & 0 & 0\\ R_{5} & 2R_{2} & R_{8} & R_{9} & R_{11} & R_{14}\\ R_{6} & R_{8} & 2R_{3} & R_{10} & R_{12} & R_{15}\\ R_{7} & R_{9} & R_{10} & 2R_{4} & R_{13} & R_{16}\\ 0 & R_{11} & R_{12} & R_{13} & 0 & 0\\ 0 & R_{14} & R_{15} & R_{16} & 0 & 0 \end{array}\right]$ and the expressions for *R*_1_–*R*_16_ are listed in Appendix A.

### 3.2. Pylon

The pylon subjected to an external load is similar to a cantilever. The deformation uses a quartic function, while the force in the pylon is in the form of a quadratic function, which is as follows:

(12) $$w_{t}=c_{1}x^{2}\left(x^{2}-4hx+6h^{2}\right)+c_{2}x^{2}\left(3h-x\right)\;$$

(13) $$M_{t}=(e_{7}x+e_{8})(x-h)\;$$

where *c*_1_, *c*_2_, *e*_7_, and *e*_8_ are the coefficients to be solved. 

Hence, the flexural strain energy of the two pylons in the Hellinger–Reissner principle is expressed as:

(14) $$\begin{array}{l} \pi_{\mathrm{HRt}}=2\int_{0}^{h}\left(-M_{t}\right)\left(\frac{d^{2}w_{t}}{dx^{2}}\right)dx-\int_{0}^{h}\frac{M_{t}{}^{2}}{E_{t}I_{t}}dx\\ \;\;\;\;\;\;\;\;\;=U_{1}e_{7}{}^{2}+U_{2}e_{8}{}^{2}+U_{3}e_{7}e_{8}+U_{4}c_{1}e_{7}+U_{5}c_{1}e_{8}+U_{6}c_{2}e_{7}+U_{7}c_{2}e_{8} \end{array}$$

where the expressions of *U*_1_ to *U*_7_ are described in Appendix A. 

### 3.3. Main Cable of the Suspension Part

The tensile strain energy of the main cable is considered here, while the flexural strain energy of the main cable is neglected. The main cable was divided into two parts during the computing procedures: the main cable crossing the main span and the main cable crossing the side span. The parts were individually investigated.

The main cable crossing the main span was assumed to be a parabola. The shape of this part is described by the following expression in a local coordinate system in which the ‘0′ in the abscissa starts from the left pylon:

(15) $$y=\frac{4f}{L_{m}}x(L_{m}-x)\;$$

where *f* is the main cable sag and *L*_m_ is the length of the main span.

**Figure 3:**
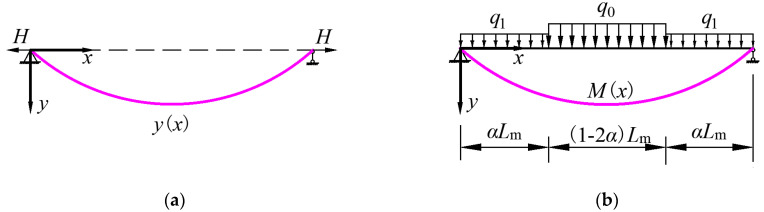
Analogy between the main cable and a simply supported beam. (**a**) Main cable subjected to load. (**b**) Equivalent simply supported beam.

The shape of the main cable is similar to the deformation of a simply supported beam subjected to a load [37], as shown in Figure 3. Hence, the following relationship can be obtained according to the analogy between the main cable and the simply supported beam:

(16) $$H\frac{d^{2}y}{dx^{2}}=-q(x)\;\;\;\;\;\;\;\;↔\;\;\;\;\;\;\frac{d^{2}M}{dx^{2}}=-q(x)\;\;$$

which gives the expression *Hy*(*x*) = *M*(*x*), where *H* is the horizontal component of the load in the main cable, *q* is the distributed load on the simply-supported beam, *M* is the bending moment distribution along the beam, *α* in Figure 3b is calculated as *α* = (*L*_1_ + *L*_2 −_ *L*_b_)/*L*_m_ = *L*_cs_/*L*_m_, where *L*_cs_ is the horizontal distance from the pylon to the first left hanger. 

Hence, the bending moment for a simply supported beam with the same span can be calculated as:

(17) $$M=\frac{1}{8}[4q_{1}\alpha^{2}L_{m}{}^{2}+(1-4\alpha^{2})q_{0}L_{m}{}^{2}]\;$$

When there is a live load on the girder, the main cable is subjected to the vertical membrane tension stress *t*_1_, as shown in Figure 2. The *q*_0_ and *q*_1_ in Figure 3b are computed as *q*_0_ *= t*_1_ and *q*_1_ *=* 0, respectively, according to which the horizontal component of the force in the main cable can be computed as:

(18) $$H=\frac{1}{8f}[(1-4\alpha^{2})t_{1}L_{m}{}^{2}]\;$$

Compared with suspension bridges, the horizontal component *H* here should be derived by multiplying *H* by a reduction factor (1 − 4*α*^2^). Consequently, the expression in the Hellinger–Reissner principle for the main cable crossing the midspan part is as follows:

(19) $$\pi_{\mathrm{mc}1}=\int_{0}^{L_{m}}\frac{{\left[H\sqrt{1+{\left(y\prime \right)}^{2}}\right]}^{2}}{E_{\mathrm{mc}}A_{\mathrm{mc}}}\sqrt{1+{\left(y\prime \right)}^{2}}dx\;\;=(1+8n^{2}+\frac{96}{5}n^{4})\frac{{\left(1-4\alpha^{2}\right)}^{2}t_{1}{}^{2}L_{m}{}^{3}}{128n^{2}E_{\mathrm{mc}}A_{\mathrm{mc}}}\;$$

The elongation of the main cable of the side-span part is dependent on the displacement of the pylon top. The displacement of the top of the pylon is expressed as *u*_t_=3*h*^4^*c*_1_+2*h*^3^*c*_2_.

The strain energy of the main cable corresponding to the left- and right-side span parts can be expressed as

(20) $$\pi_{\mathrm{mc}2}=2\times \frac{1}{2}F_{\mathrm{mc}}\Delta =2\times \frac{1}{2}\cdot \frac{E_{\mathrm{mc}}A_{\mathrm{mc}}u_{t}\cos\varphi }{L_{b}/\cos\varphi }\cdot u_{t}\cos\varphi \;\;=G_{1}c_{1}{}^{2}+G_{2}c_{2}{}^{2}+G_{3}c_{1}c_{2}\;$$

where *E*_mc_ is the Young’s modulus of the main cable, *A*_mc_ is the cross-sectional area of the main cable, *n* is the ratio of the main cable sag to the span, which is calculated as *n* = *f*/*L*_m_, *F*_mc_ is the axial force in the main cable of the side-span part, Δ is the elongation of the main cable, and *φ* is the angle between the girder and the main cable in the side span.

Hence, by combining Equations (19) and (20), the total strain energy of the main cable in the Hellinger–Reissner principle can be described as:

(21) $$\pi_{\mathrm{mc}}=\pi_{\mathrm{mc}1}+\pi_{\mathrm{mc}2}=G_{1}c_{1}{}^{2}+G_{2}c_{2}{}^{2}+G_{3}c_{1}c_{2}+G_{4}t_{1}{}^{2}\;$$

where the expressions for *G*_1_ to *G*_4_ are listed in Appendix A. 

### 3.4. External Load Effects on the Bridge

According to the simplified mechanical model shown in Figure 2, the work done by the external load can be divided into the following parts: (1) the live load effect on the girder, (2) the membrane tensile load *t*_1_ caused by the hangers on the girder, (3) the membrane tensile load *t*_1_ from the hangers on the main cable, (4) the membrane tensile loads *t*_2_ and *t*_3_ from the stay cables on the girder, and (5) the membrane tensile loads *t*_2T_ and *t*_3T_ from the stay cables on the pylons. To simplify the computing procedures, an analysis of the work done by the external load was carried out in the global coordinate system.

(1) Effects of live load on girder:

The live loads on the bridge could be the different combinations of *q* and *P* in Figure 2. Three load combinations are taken here as examples to show the expressions of the potential energy of the external load in the Hellinger–Reissner method. In addition, the different load combinations help to determine whether the hypotheses of the trial functions are appropriate. 

In the case of the first type of load combination, the entire bridge is loaded with a uniformly distributed load *q*. The potential energy of the external load is expressed as:

(22) $$\begin{array}{l} \pi_{q1}=-2\int_{0}^{L_{c}}qw_{1}dx-\int_{0}^{L_{s}}qw_{2}dx\\ \;\;\;\;\;\;\;=b_{1}\left(L_{1}{}^{3}L_{c}{}^{2}-\frac{2L_{c}{}^{5}}{5}\right)q+b_{2}\left(L_{1}{}^{2}L_{c}{}^{2}-\frac{L_{c}{}^{4}}{2}\right)q-\frac{1}{30}a_{1}L_{s}{}^{5}q-\frac{1}{5}a_{2}L_{s}{}^{5}q-a_{3}L_{s}q\\ \;\;\;\;\;\;\;=Q_{11}b_{1}+Q_{12}b_{2} \end{array}$$

In the second case of load combination, only the main span of the bridge is loaded with a uniformly distributed load *q*. Hence, the potential energy by the external load can be expressed as:

(23) $$\begin{array}{l} \pi_{q2}=-2\int_{L_{b}}^{L_{c}}qw_{1}dx-\int_{0}^{L_{s}}qw_{2}dx\\ \;\;\;\;\;\;\;=b_{1}\left[L_{1}{}^{3}(L_{c}{}^{2}-L_{b}{}^{2})-\frac{2(L_{c}{}^{5}-L_{b}{}^{5})}{5}\right]q+b_{2}\left[L_{1}{}^{2}(L_{c}{}^{2}-L_{b}{}^{2})-\frac{\left(L_{c}{}^{4}-L_{b}{}^{4}\right)}{2}\right]q\\ \;\;\;\;\;\;\;\;\;\;\;\;-\frac{1}{30}a_{1}L_{s}{}^{5}q-\frac{1}{5}a_{2}L_{s}{}^{5}q-a_{3}L_{s}q\\ \;\;\;\;\;\;\;=Q_{21}b_{1}+Q_{22}b_{2} \end{array}$$

The third type of load condition is the uniformly distributed load *q* on the girder corresponding to the suspension part of the bridge. The expression of potential energy by the external load is:

(24) $$\pi_{q3}=-\int_{0}^{L_{s}}qw_{2}dx\;\;=-\frac{1}{30}a_{1}L_{s}{}^{5}q-\frac{1}{5}a_{2}L_{s}{}^{5}q-a_{3}L_{s}q\;\;=Q_{31}b_{1}+Q_{32}b_{2}\;$$

The following term of the effect caused by the concentrated load *P* can be added to the abovementioned expressions, Equations (22)–(24), if the concentrated load is considered:

(25) $$\pi_{p}\;=-\frac{1}{16}a_{1}L_{s}{}^{4}P-\frac{5}{16}a_{2}L_{s}{}^{4}P-a_{3}P\;\;=Q_{p1}b_{1}+Q_{p2}b_{2}\;$$

where the coefficients *Q*_11_, *Q*_12_, *Q*_21_, *Q*_22_, *Q*_31_, *Q*_32_, *Q*_p1_, and *Q*_p2_ are shown in Appendix A.

(2) Effect of membrane tension *t*_1_ on girder:

The work done by the hanger membrane tension on the girder is negative, assuming that the positive displacement of the girder is vertically downward and the membrane tension on the girder is vertically upward.

(26) $$\begin{array}{l} \pi_{2}=\int_{0}^{L_{s}}t_{1}w_{2}dx+2\int_{L_{x}}^{L_{c}}t_{1}w_{11}dx\\ \;\;\;\;\;=\left[\frac{2}{5}\left(L_{c}{}^{5}-L_{x}{}^{5}\right)-L_{1}{}^{3}\left(L_{c}{}^{2}-L_{x}{}^{2}\right)\right]b_{1}t_{1}+\left[\frac{1}{2}\left(L_{c}{}^{4}-L_{x}{}^{4}\right)-L_{1}{}^{2}\left(L_{c}{}^{2}-L_{x}{}^{2}\right)\right]b_{2}t_{1}\\ \;\;\;\;\;+\frac{1}{30}a_{1}L_{s}{}^{5}t_{1}+\frac{1}{5}a_{2}L_{s}{}^{5}t_{1}\;\;\;\;\;+a_{3}L_{s}t_{1}\\ \;\;\;\;\;=D_{1}b_{1}t_{1}+D_{2}b_{2}t_{1} \end{array}$$

where *L*_x_ is equal to (*L*_1_ + *L*_2_), and *D*_1_ and *D*_2_ are listed in Appendix A.

(3) Effect of membrane tension *t*_1_ on main cable:

The displacement at the midspan of the main cable is Δ*f*. The main cable under the load maintains a parabolic shape. Hence, the expression for the shape of the main cable after deformation is expressed as:

(27) $$y_{2}=\frac{4(f+\Delta f)}{L_{m}}x(L_{m}-x)\;$$

The deformation of the main cable at midspan under the live load is related to the deformation of the pylon top and elongation of the main cable. The two parts of the deformation are independent of each other. The effects of these two parts on the deformation of the main cable were derived by Chai et al. [37]:

(28) $$\frac{\Delta f_{1}}{2u_{t}}=\frac{1-\frac{8}{3}n^{2}+\frac{96}{5}n^{4}}{\frac{16}{3}n-\frac{128}{5}n^{3}}$$

(29) $$\frac{\Delta f_{2}}{\Delta l_{\mathrm{mc}}}=\frac{1}{\frac{16}{3}n-\frac{128}{5}n^{3}}$$

where Δ*f*_1_ is the deformation of the main cable caused by the displacement of the pylon top, Δ*f*_2_ is the deformation of the main cable caused by the elongation of the main cable itself, and Δ*l*_mc_ is the amount of elongation of the main cable, expressed as:

(30) $$\Delta l_{\mathrm{mc}}=\int_{0}^{L_{m}}\frac{H\sqrt{1{+(y\prime )}^{2}}}{E_{\mathrm{mc}}A_{\mathrm{mc}}}\sqrt{1{+(y\prime )}^{2}}dx\;$$

which provides Δ*f* = Δ*f*_1_ + Δ*f*_2_.

The expression of the work done by membrane tension *t*_1_ on the main cable is:

(31) $$\pi_{3}=-\int_{L_{\mathrm{cs}}}^{L_{m}-L_{\mathrm{cs}}}t_{1}(y_{2}-y)dx\;\;\;=\left(-\frac{2}{3}L_{0}+\frac{4L_{\mathrm{cs}}{}^{2}}{L_{0}}-\frac{8L_{\mathrm{cs}}{}^{3}}{3L_{0}{}^{2}}\right)\Delta ft_{1}=D_{3}c_{1}t_{1}+D_{4}c_{2}t_{1}+D_{5}t_{1}{}^{2}\;$$

where the coefficients *D*_3_ to *D*_5_ are shown in Appendix A.

(4) Effects of membrane tensions *t*_2_ and *t*_3_ on girder:

The calculation of the effects of stay cable membrane tensions *t*_2_ and *t*_3_ on the girder is similar to that of the hanger membrane tension on the girder and main cable. Hence, the expression for the effects of *t*_2_ and *t*_3_ on the girder can be expressed as:

(32) $$\pi_{4}=2\int_{0}^{L_{b}}t_{3}w_{11}dx+2\int_{L_{b}}^{L_{c}}t_{2}w_{11}dx\;\;=D_{6}b_{1}t_{2}+D_{7}b_{1}t_{3}+D_{8}b_{2}t_{2}+D_{9}b_{2}t_{3}\;$$

where the coefficients *D*_6_ to *D*_9_ are shown in Appendix A.

**Figure 4:**
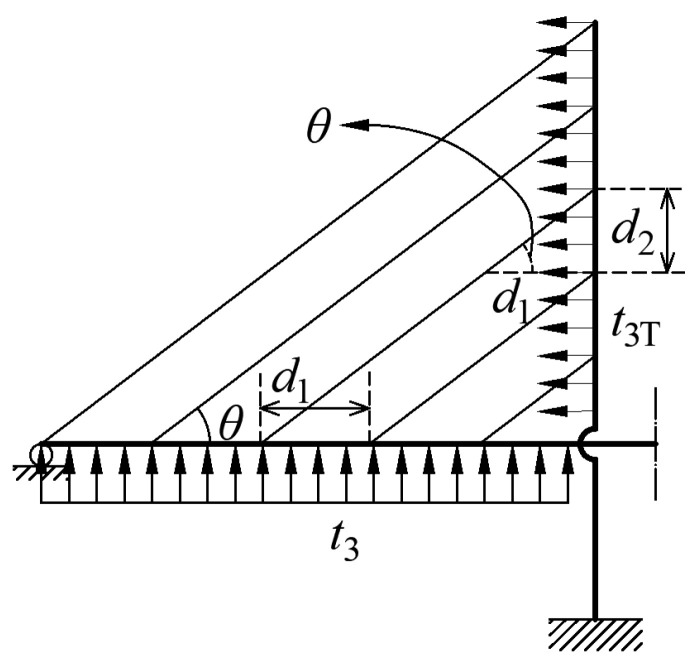
Parameters of membrane tension for the stay cable.

(5) Effects of membrane tensions *t*_2T_ and *t*_3T_ on the pylon:

It can be observed from Figure 4 that the relationship between the stay cable membrane tension on the girder and that on the pylon can be expressed as:

(33) $$t_{2T}=\frac{t_{2}\cot\theta d_{1}}{d_{2}}\;$$

(34) $$t_{3T}=\frac{t_{3}\cot\theta d_{1}}{d_{2}}\;$$

where *θ* is the stay cable inclination angle, as shown in Figure 4, *d*_1_ is the space between the stay cables on the girder, *d*_2_ is the space between the stay cables on the pylon, and the relationship between the three factors is cot(*θ*) = *d*_1_*/d*_2_ if the stay cables are parallel to each other. 

The stay cable membrane tensions are uniformly distributed along the pylon. The work done by the membrane tensions *t*_2T_ and *t*_3T_ on the pylon is expressed as:

(35) $$\pi_{5}=-2\int_{h_{c1}}^{h_{c2}}(t_{2}-t_{3}){\left(\cot\theta \right)}^{2}w_{t}dx\;\;=D_{10}c_{1}t_{2}+D_{11}c_{1}t_{3}+D_{12}c_{2}t_{2}+D_{13}c_{2}t_{3}\;$$

where *h*_c1_ and *h*_c2_ are the heights of the beginning and end points of the stay cables anchored on the pylon, which are equal to *h*_1_ and *h*, respectively, as shown in Figure 3, if the stay cable membrane tension is uniformly distributed along the pylon above the girder; the coefficients *D*_10_ to *D*_13_ are shown in Appendix A. 

### 3.5. Total Potential Energy

According to each part of the potential energy explained above, the total potential energy of the system can be obtained as:

(36) $$\pi_{\mathrm{HR}}=\pi_{\mathrm{HRb}}+\pi_{\mathrm{HRt}}+\pi_{\mathrm{mc}}+\pi_{\mathrm{qi}}+\pi_{p}+\pi_{2}+\pi_{3}+\pi_{4}+\pi_{5}\;$$

where *π_qi_* is the work done by the *i*_th_ type of load condition on the girder.

(37) $$\frac{\partial \pi_{\mathrm{HR}}}{\partial \xi_{i}}=0\;$$

where *ξ_i_* is the *i*_th_ element in the vector *ξ*_HR_, and *ξ*_HR_ is expressed as:

$$\xi_{HR}=[\begin{array}{cccccccccc} e_{1} & e_{2} & e_{3} & e_{5} & b_{1} & b_{2} & c_{1} & c_{2} & e_{7} & e_{8} \end{array}{]}^{T}$$

To sum up, Equation (37) can be transformed into the following matrix equation:

(38) $$K_{HR}\xi_{HR}=F\;$$

where **K_HR_** and **F** could be expressed as:

$$K_{\mathrm{HR}}=\left[\begin{array}{cccccccccc} 2R_{1} & R_{5} & R_{6} & R_{7} & 0 & 0 & 0 & 0 & 0 & 0\\ & 2R_{2} & R_{8} & R_{9} & R_{11} & R_{14} & 0 & 0 & 0 & 0\\ & & 2R_{3} & R_{10} & R_{12} & R_{15} & 0 & 0 & 0 & 0\\ & & & 2R_{4} & R_{13} & R_{16} & 0 & 0 & 0 & 0\\ & & & & 0 & 0 & 0 & 0 & 0 & 0\\ & & & & & 0 & 0 & 0 & 0 & 0\\ & & \mathrm{sym} & & & & 2G_{1} & G_{3} & U_{4} & U_{5}\\ & & & & & & & 2G_{2} & U_{6} & U_{7}\\ & & & & & & & & 2U_{1} & U_{3}\\ & & & & & & & & & 2U_{2} \end{array}\right]F=\left[\begin{array}{c} 0\\ 0\\ 0\\ 0\\ -Q_{i1}-Q_{p1}-D_{1}t_{1}-D_{6}t_{2}-D_{7}t_{3}\\ -Q_{i2}-Q_{p2}-D_{2}t_{1}-D_{8}t_{2}-D_{9}t_{3}\\ -D_{3}t_{1}-D_{10}t_{2}-D_{11}t_{3}\\ -D_{4}t_{1}-D_{12}t_{2}-D_{13}t_{3}\\ 0\\ 0 \end{array}\right]$$

### 3.6. Deformation Compatibility Conditions

**Figure 5:**
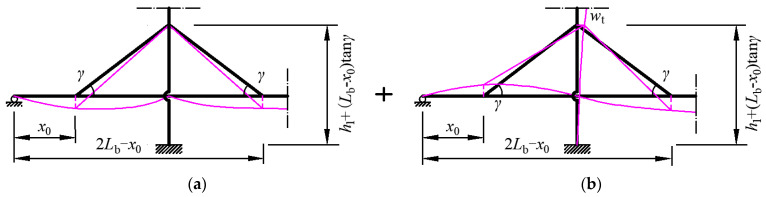
Deformation compatibility conditions between the stay cables and girder. (**a**) Displacement caused by elongation of stay cables. (**b**) Displacement caused by pylon deformation.

The expressions for the coefficients can be obtained by solving Equation (38), but as a function of *t*_1_, *t*_2_, and *t*_3_. However, solutions for the coefficients have not yet been obtained at this step. Hence, deformation compatibility conditions shown in Figure 5 are introduced to derive the solutions for the coefficients.

The first deformation compatibility condition in Figure 5a is that the midspan displacement of the girder is equal to that of the main cable if the elongation of the hanger is neglected.

(39) $$\Delta f=\frac{1}{16}L_{s}{}^{4}a_{1}+\frac{5}{16}L_{s}{}^{4}a_{2}+a_{3}\;$$

The other deformation compatibility condition shown in Figure 5b is that the displacement of the stay cable is the same as the displacement of the girder at the point where the stay cable is anchored to the girder. Meanwhile, the stay cable displacement at this point is related to the elongation itself and the horizontal deformation of the pylon. These two deformation compatibility conditions are expressed as follows:

(1) Side span:

(40) $$\frac{t_{3}d_{1}(L_{b}-x_{0})}{E_{c}A_{c}\sin^{2}\gamma \cos\gamma }-{\left.w_{t}\right|}_{x=h_{1}+(L_{b}-x_{0})\tan\gamma }={\left.w_{b}\right|}_{x=x_{0}}\;$$

(2) Main span:

(41) $$\frac{t_{2}d_{1}(L_{b}-x_{0})}{E_{c}A_{c}\sin^{2}\gamma \cos\gamma }+{\left.w_{t}\right|}_{x=h_{1}+(L_{b}-x_{0})\tan\gamma }={\left.w_{b}\right|}_{x=2L_{b}-x_{0}}\;$$

where *x*_0_ is the location on the abscissa of the stay cable anchored at the girder, and *γ* is the inclination angle of the stay cable, which is equal to *θ* when the stay cables are parallel to each other. If the sag effect of the stay cables is considered, the Young’s modulus of the stay cables can be adjusted or reduced according to the Ernst equation [38] if the initial tension of the stay cables is already known.

The membrane tension can be solved using Equations (39)–(41). Based on this, the solutions for the deformation of the structure members can be presented. 

## 4. Verification

**Figure 6:**
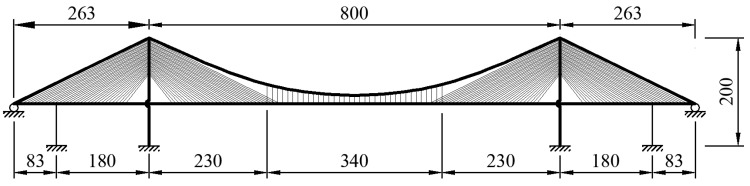
Dimensional properties of the test model of the bridge (unit: m).

To carry out the verification of the presented method, the results from the finite element analysis and test are both compared with the presented H-R method. First, a cable-stayed suspension bridge with a 263 + 800 + 263 m span is introduced here [18,19]. The geometric parameters are presented in Figure 6. The pylon was made of Chinese C50 concrete. The total height of the pylon is 200 m. The girder of the cable-stayed part is also made of Chinese C50 concrete with a rectangular cross-section, whereas the girder of the suspension part is made of an aluminum alloy box. Meanwhile, the tensile strength value of both the stay cable and the main cable is 1600 MPa. The space between stay cables along the girder is 16 m, while the space along the pylon is 2–3 m. The space between the hangers along the girder is also 16 m. There is one auxiliary pier supporting each side span. Hence, a fully floating deck system is applied here, which means that the pylon and girder are not connected longitudinally along the girder or vertically along the pylon. 

Researchers [18,19] have studied the structural behavior of this bridge by building a 1:100 bridge model in the laboratory. Both the finite element results and test results along with more detailed information are provided in the literature [18,19]. The software used in the finite element analysis is ANSYS. Both the beam and pylon were using the 3D BEAM4 elements. The hangers and the stay cables were using the LINK10 elements, which were set to be subjected to tensile force only. The geometric nonlinear analyses were performed. 

The key parameters of the structural members are listed in Table 1. 

**Figure 7:**
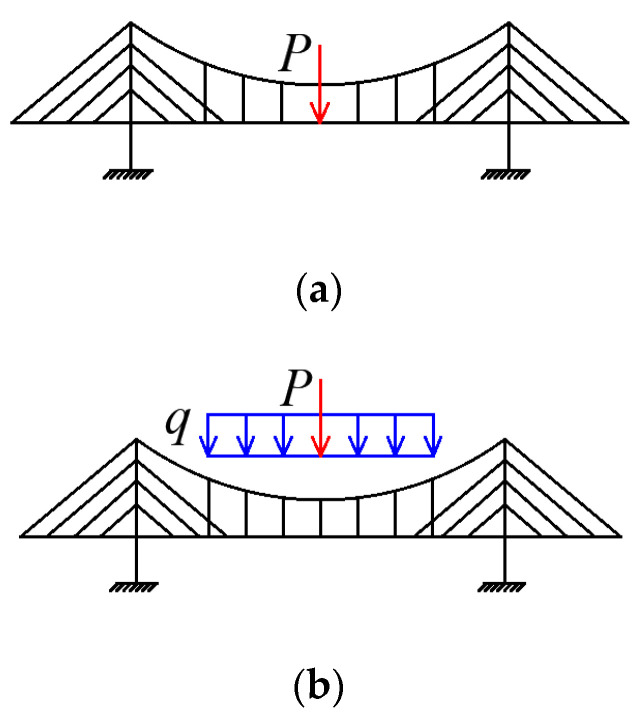

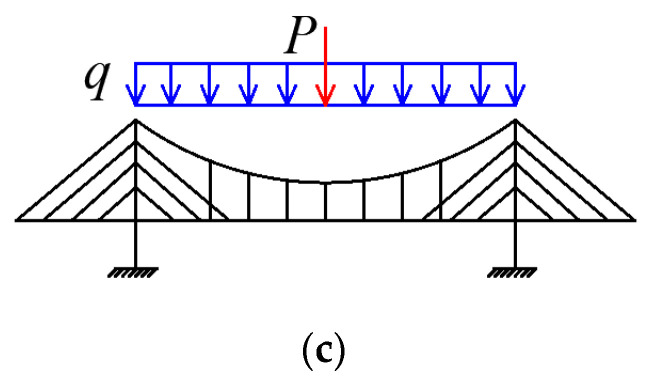
Load combinations. (**a**) Load combination 1 (*P* = 500 N). (**b**) Load combination 2 (*P* = 250 N, *q* = 125 N/m). (**c**) Load combination 3 (*P* = 250 N, *q* = 125 N/m).

The midspan deflection and the displacement of the top of the pylon were investigated using both tests and the finite element analysis by Du [18] and Wang [19]. The results are compared with the numerical data from the Hellinger–Reissner method. There are three types of load combinations in the comparison analysis. The first load combination, called load combination 1 here, involves a concentrated force P = 500 N on the midspan, as shown in Figure 7a. Another type of load combination, called load combination 2, is shown in Figure 7b and includes a concentrated force *P* = 250 N on the midspan and a distributed load *q* = 125 N/m along the suspension part. Figure 7c shows the third load combination, called load combination 3. It is composed of a concentrated force *P* = 250 N on the midspan and a distributed load *q* = 125 N/m along the main span.

**Figure 8:**
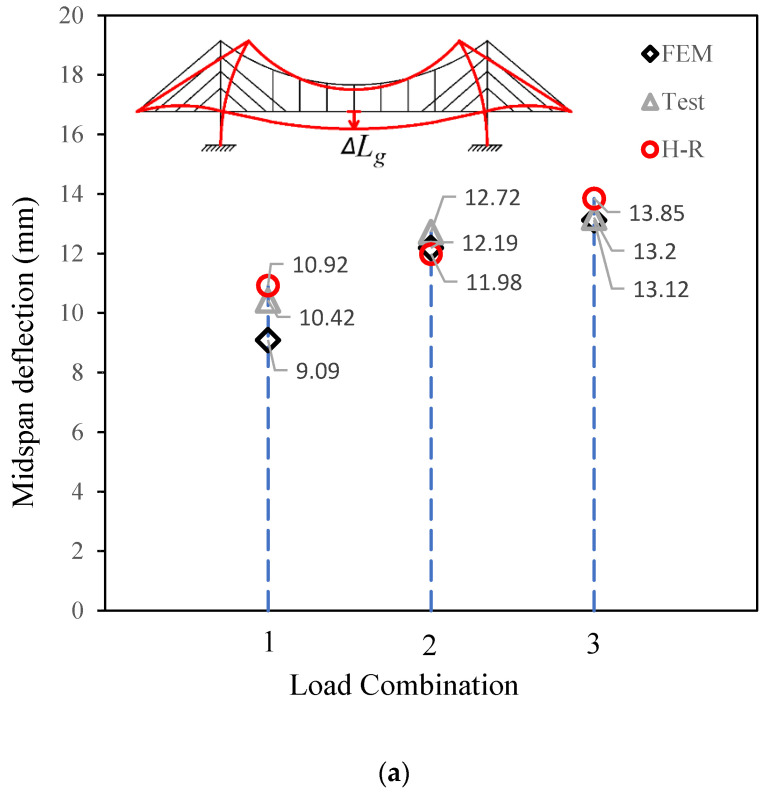

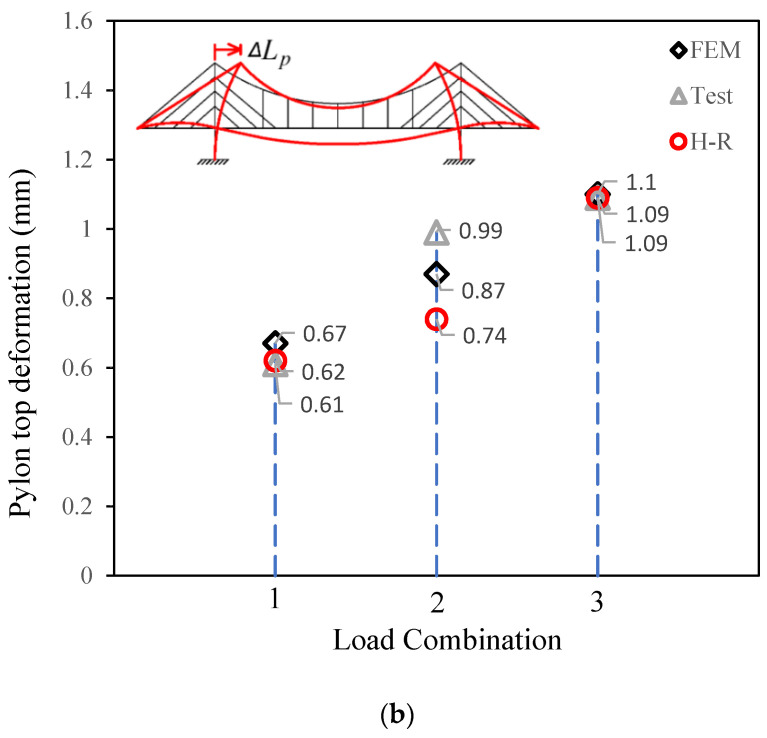
Comparisons between different methods. (**a**) Comparison of midspan deflections. (**b**) Comparison of the pylon top deformation.

The comparison results are then shown in Figure 8. The ordinate of Figure 8a is the midspan deflection, while the ordinate in Figure 8b is the displacement of the top of the pylon. The positive direction is indicated by arrows in the figures.

Figure 8a shows that the fitting among the results from the three methods is good for midspan deflection. The maximum gap is between the results from the Hellinger–Reissner method and the finite element method, with an error of 10% under load combination 1. Meanwhile, for the same type of load combination, the error between the test and the Hellinger–Reissner method is less than 5%. 

For the top displacement of the pylon in Figure 8b, the maximum error, which is approximately 20%, occurs under load combination 2. The other two load combinations were in good agreement among the three types of methods. One of the reasons for this may be that the absolute values are small, which makes the comparative error much larger. The other reason could be explained as maybe there are some errors in the operation of the test based on the fact that the error between the finite element analysis and tests is also large. The third reason could be the hypothesis of deformation expression. The conduction of the H-R method requires the hypothesis of both the deformation expression and the force expression. Engineers should have certain experience to decide which expression to use at the early stage of analysis based on the H-R method. In addition, simplified evaluation would lead to errors in the results. 

## 5. Sensitivity Analyses

**Figure 9:**
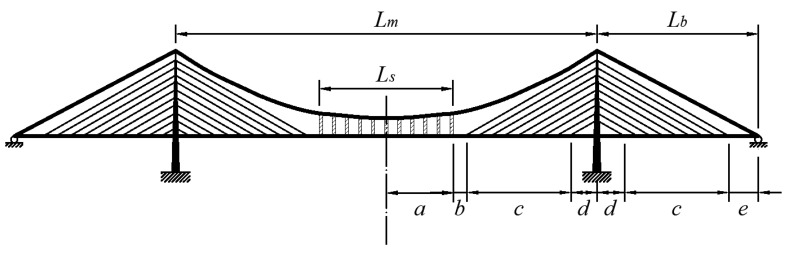
Arrangement of dimensional properties.

The impact of the sag-to-span ratio on the deformation of the combined cable-stayed suspension bridge was then investigated. Jin et al. [39] provided the results from the finite element analysis of the deflection at the midspan and the displacement of the top of the pylon. The Hellinger–Reissner method was applied here with the same material properties and the same load condition mentioned in a published paper [39]. The corresponding data were obtained and compared with the finite element analysis. The span arrangement is illustrated in Figure 9. The length of the main span *L*_m_ is equal to twice the summation of ‘a’, ‘b’, ‘c’, and ‘d’. The side span *L*_b_ is equal to the summation of ‘d’, ‘c’, and ‘e’.

Detailed values are listed in Table 2. There are five different types of span arrangements. The different types are named ‘A’, ‘B’, C’, ‘D’, and ‘E’. The sag-to-suspension ratio listed in the fifth column of Table 2 varies from 0.4 to 0.8. 

**Figure 10:**
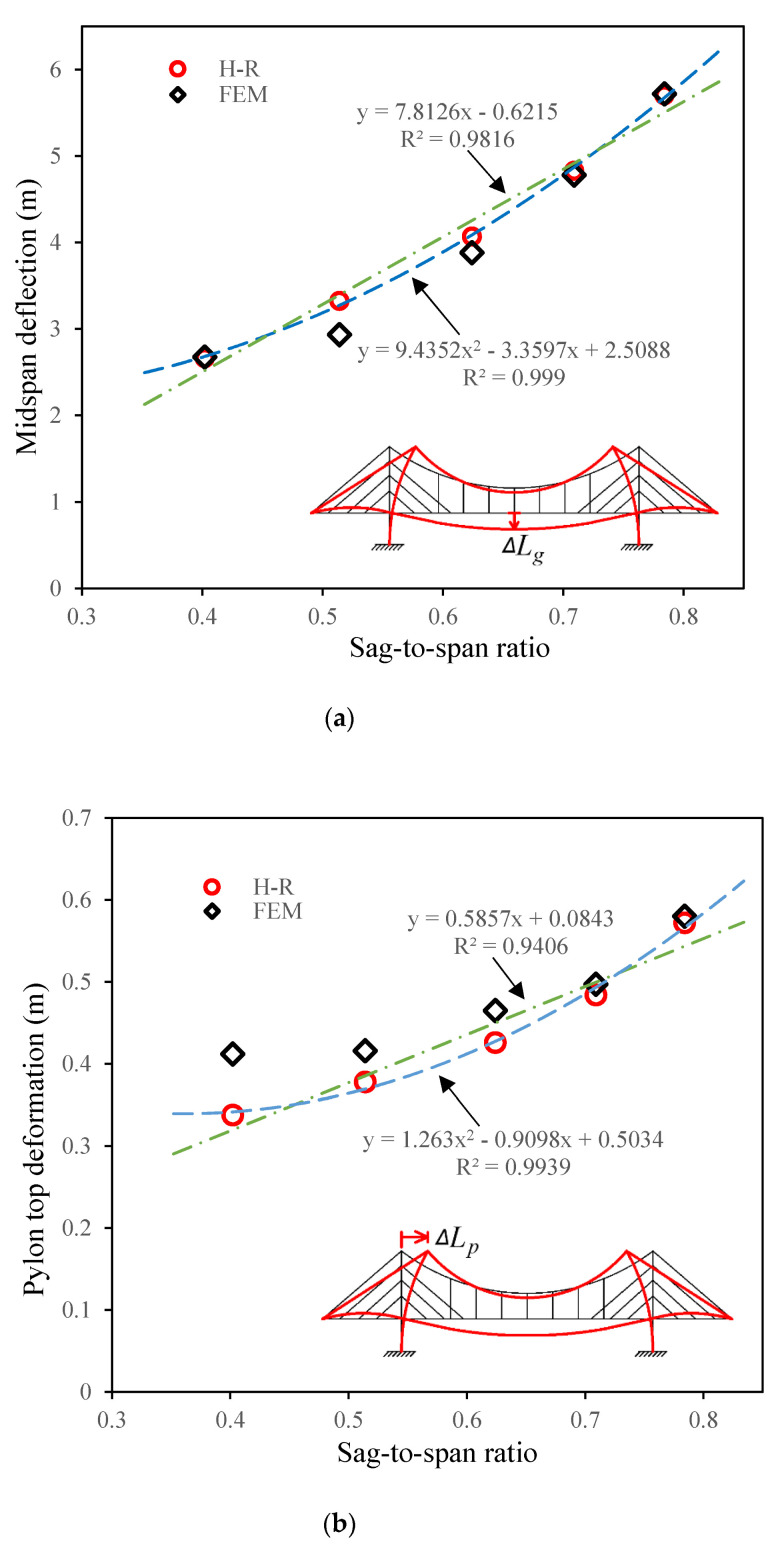
Impact of sag-to-span ratio on deformation. (**a**) Midspan deflection changing with sag-to-span ratio. (**b**) Pylon top deformation changing with sag-to-span ratio.

As illustrated in Figure 10a, the comparison shows that the fitting of the midspan deflection is relatively good from the sag-to-suspension ratio of 0.4 to 0.8. The values of *ΔL_g_* from the two methods are almost the same for models A, D, and E, as shown in Figure 10a. The errors between the two methods are approximately 10% and 5%, respectively, in models B and C. The data points obtained from the Hellinger–Reissner method were fitted using linear and quadratic equations. As shown in Figure 10a, the r-square values for both expressions are close to 1. This also means that the distribution of the data points is in a very clear form of linear ascending. 

The fitting of the top displacement of the pylon, *ΔL_p_*, is shown in Figure 10b. The errors between the Hellinger–Reissner method and the finite element analysis imply that the increasing of sag-to-suspension ratio improves the fitting between the two methods. The maximum discrepancy exists in the case of model A, where the error reaches 17%. It shows that the data points of the Hellinger–Reissner method were also fitted by the linear and quadratic equations with good fitting results. 

## 6. Conclusions

The Hellinger–Reissner variational principle is introduced here to investigate the structural behavior of the combined cable-stayed suspension bridge. The procedures of the application of Hellinger–Reissner variational principle in the analysis of cable-stayed suspension bridge is described in detail, which consist of the hypotheses of the deformation, the hypotheses of the force expressions for the structural members, along with the boundary compatibility conditions to build the matrix equation. By solving the matrix equation, the coefficients of the deformation and force expressions are derived directly. The main works are described as follows:

(1) One case of the cable-stayed suspension bridge was analyzed and compared with finite element analysis and test results, and the comparisons showed good agreement. This means that the results from the Hellinger–Reissner method satisfy the requirements of the conceptual design stage. 

(2) Parametric discussion including the sag-to-span ratio of the bridge was conducted, showing that the agreement between the Hellinger–Reissner results and the finite element analysis increases as the sag-to-span ratio goes up within the appropriate range. 

(3) The Hellinger–Reissner method is efficient in solving the deformation and force of the members of bridges with a combined system. It could be used as a quick solution to help designers make decisions in the early design stage. Besides, it could also be used to verify the finite element analysis to reduce human errors. 

## 7. Outlook

This article provides methodology for the early design stage of the combined cable-stayed suspension bridge. However, this methodology could be extended to some complicated problems during the state of serviceability, e.g., dynamic behavior, geometric nonlinearities of the suspension cables, and torsional effects. These problems could not be neglected when the bridge is in service state.

## Figures and Tables

**Table 1:** Material properties.

Structural Member	*E* (MPa)	Dimension
Girder (concrete part)	3.45 × 10^4^	*I* (m^4^) = 1.883 × 10^−6^
Girder (aluminum alloy part)	7.0 × 10^4^	*I* (m^4^) = 4.819 × 10^−7^
Pylon	3.45 × 10^4^	*I* (m^4^) = 5.0 × 10^−6^
Main cable	2.05 × 10^5^	*A* (m^2^) = 7.854 × 10^−7^
Stay cable	2.05 × 10^5^	*A* (m^2^) = 1.964 × 10^−7^

**Table 2:** Different dimensional arrangement.

Model	Main Span (m) 2 × (a + b + c + d)	Side Span (m) d + c + e	Side Span/Main Span *L_b_*/*L_m_*	Sag-to-Span Ratio *f*/*L*_m_
A	2 × (28 × 9 + 13 + 39 × 9 + 34) = 1300	34 + 39 × 9 + 10 = 395	0.304	0.402
B	2 × (39 × 9 + 18 + 33 × 9 + 34) = 1400	34 + 33 × 9 + 14 = 345	0.246	0.514
C	2 × (51 × 9 + 14 + 27 × 9 + 34) = 1500	34 + 27 × 9 + 18 = 295	0.197	0.624
D	2 × (62 × 9 + 10 + 22 × 9 + 34) = 1600	34 + 22 × 9 + 13 = 245	0.153	0.709
E	2 × (73 × 9 + 15 + 16 × 9 + 34) = 1700	34 + 16 × 9 + 17 = 195	0.115	0.784

## Nomenclature

*A*	cross-section area
*d*	distance between stay cables
*E*	Young’s modulus
*f*	rise of the main cable
*h*	height of the pylon
I	inertia moment
*M*	function for bending moment
*L*_b_	length of the side span
*L*_m_	length of the main span
L_s_	length of the suspension part
*P*	concentrated load on the bridge
*q*	uniformly distributed load on the bridge
*t*_1_	membrane tension of hangers
*t*_2_	membrane tension of stay cable on main span
*t*_3_	membrane tension of stay cable on side span
*w*	function for deflection
Δ*f*	elongation of the hanger
π	function for potential energy
θ	inclination angle of the stay cable

## Appendix A

The following expressions are provided for describing the procedures of the methodology.

***Expressions for R_1_–R_16_:***

$$\begin{array}{l} R_{1}=-\frac{L_{1}{}^{3}}{3E_{b1}I_{b1}}-\frac{L_{1}{}^{2}L_{3}}{E_{b1}I_{b1}}-\frac{L_{1}{}^{2}L_{s}}{2E_{b2}I_{b2}},\;R_{2}=-\frac{L_{1}}{E_{b1}I_{b1}}-\frac{L_{3}}{E_{b1}I_{b1}}-\frac{L_{s}}{2E_{b2}I_{b2}}R_{3}=-\frac{L_{3}{}^{3}}{3E_{b1}I_{b1}}-\frac{L_{3}{}^{2}L_{s}}{2E_{b2}I_{b2}},\;R_{4}=-\frac{L_{s}{}^{5}}{60E_{b2}I_{b2}},\;\\ R_{5}=-\frac{L_{1}{}^{2}}{E_{b1}I_{b1}}-\frac{2L_{1}L_{3}}{E_{b1}I_{b1}}-\frac{L_{1}L_{s}}{E_{b2}I_{b2}},\;R_{6}=-\frac{L_{1}L_{3}{}^{2}}{E_{b1}I_{b1}}-\frac{L_{1}L_{3}L_{s}}{E_{b2}I_{b2}},\;R_{7}=\frac{L_{1}L_{s}{}^{3}}{6E_{b2}I_{b2}},\;R_{8}=-\frac{L_{3}{}^{2}}{E_{b1}I_{b1}}-\frac{L_{3}L_{s}}{E_{b2}I_{b2}},\;R_{9}=\frac{L_{s}{}^{3}}{6E_{b2}I_{b2}}\\ R_{10}=\frac{L_{3}L_{s}{}^{3}}{6E_{b2}I_{b2}},\;R_{11}=-2L_{1}{}^{3},\;R_{12}=6L_{1}{}^{3}L_{3}+12L_{1}{}^{2}L_{3}{}^{2}+8L_{1}L_{3}{}^{3}+2L_{3}{}^{4},\;\\ R_{13}=-\frac{6}{5}L_{1}{}^{3}L_{s}{}^{2}-\frac{24}{5}L_{1}{}^{2}L_{3}L_{s}{}^{2}-\frac{24}{5}L_{1}L_{3}{}^{2}L_{s}{}^{2}-\frac{8L_{3}{}^{3}L_{s}{}^{2}}{5}-\frac{2L_{1}{}^{2}L_{s}{}^{3}}{5}-\frac{4}{5}L_{1}L_{3}L_{s}{}^{3}-\frac{2L_{3}{}^{2}L_{s}{}^{3}}{5},\;\\ R_{14}=-2L_{1}{}^{2},\;R_{15}=4L_{1}{}^{2}L_{3}+6L_{1}L_{3}{}^{2}+2L_{3}{}^{3},\;\\ R_{16}=-\frac{4}{5}L_{1}{}^{2}L_{s}{}^{2}-\frac{12}{5}L_{1}L_{3}L_{s}{}^{2}-\frac{6L_{3}{}^{2}L_{s}{}^{2}}{5}-\frac{L_{1}L_{s}{}^{3}}{5}-\frac{L_{3}L_{s}{}^{3}}{5}. \end{array}$$

***Expressions for U*_1_–*U*_7_:**

$$U_{1}=-\frac{h^{5}}{30E_{t}I_{t}},U_{2}=-\frac{h^{3}}{3E_{t}I_{t}},\;U_{3}=-\frac{h^{4}}{6E_{t}I_{t}},\;U_{4}=\frac{6}{5}h^{5},\;U_{5}=6h^{4},\;U_{6}=h^{4},\;U_{7}=4h^{3}$$

***Expressions for G*_1_–*G*_4_:**

$$G_{1}=\frac{9E_{\mathrm{mc}}A_{\mathrm{mc}}h^{8}\cos\varphi^{3}}{L_{b}},\;G_{2}=\frac{4E_{\mathrm{mc}}A_{\mathrm{mc}}h^{6}\cos\varphi^{3}}{L_{b}},\;G_{3}=\frac{12E_{\mathrm{mc}}A_{\mathrm{mc}}h^{7}\cos\varphi^{3}}{L_{b}},\;G_{4}=(1+8n^{2}+\frac{96}{5}n^{4})\frac{{\left(1-4\alpha^{2}\right)}^{2}L_{m}{}^{3}}{128n^{2}E_{\mathrm{mc}}A_{\mathrm{mc}}}.$$

***Expressions for Q*_11_–*Q*_32_:**

$$\begin{array}{l} Q_{11}=\left(L_{1}{}^{3}L_{c}{}^{2}-\frac{2L_{c}{}^{5}}{5}+L_{1}{}^{3}L_{c}L_{s}-L_{c}{}^{4}L_{s}+\frac{L_{1}{}^{3}L_{s}{}^{2}}{5}-\frac{4L_{c}{}^{3}L_{s}{}^{2}}{5}-\frac{L_{c}{}^{2}L_{s}{}^{3}}{5}\right)q,\;\\ Q_{12}=\left(L_{1}{}^{2}L_{c}{}^{2}-\frac{L_{c}{}^{4}}{2}+L_{1}{}^{2}L_{c}L_{s}-L_{c}{}^{3}L_{s}+\frac{L_{{}_{1}}{}^{2}L_{s}{}^{2}}{5}-\frac{3L_{c}{}^{2}L_{s}{}^{2}}{5}-\frac{L_{c}L_{s}{}^{3}}{10}\right)q,\;\\ Q_{21}=\left(-L_{1}{}^{3}L_{b}{}^{2}+\frac{2L_{b}{}^{5}}{5}+L_{1}{}^{3}L_{c}{}^{2}-\frac{2L_{c}{}^{5}}{5}+L_{1}{}^{3}L_{c}L_{s}-L_{c}{}^{4}L_{s}+\frac{L_{1}{}^{3}L_{s}{}^{2}}{5}-\frac{4L_{c}{}^{3}L_{s}{}^{2}}{5}-\frac{L_{c}{}^{2}L_{s}{}^{3}}{5}\right)q,\;\\ Q_{22}=\left(-L_{1}{}^{2}L_{b}{}^{2}+\frac{L_{b}{}^{4}}{2}+L_{1}{}^{2}L_{c}{}^{2}-\frac{L_{c}{}^{4}}{2}+L_{1}{}^{2}L_{c}L_{s}-L_{c}{}^{3}L_{s}+\frac{L_{1}{}^{2}L_{s}{}^{2}}{5}-\frac{3L_{c}{}^{2}L_{s}{}^{2}}{5}-\frac{L_{c}L_{s}{}^{3}}{10}\right)q,\;\\ Q_{31}=\left(L_{1}{}^{3}L_{c}L_{s}-L_{c}{}^{4}L_{s}+\frac{L_{1}{}^{3}L_{s}{}^{2}}{5}-\frac{4L_{c}{}^{3}L_{s}{}^{2}}{5}-\frac{L_{c}{}^{2}L_{s}{}^{3}}{5}\right)q,\;\\ Q_{32}=\left(L_{1}{}^{2}L_{c}L_{s}-L_{c}{}^{3}L_{s}+\frac{L_{1}{}^{2}L_{s}{}^{2}}{5}-\frac{3L_{c}{}^{2}L_{s}{}^{2}}{5}-\frac{L_{c}L_{s}{}^{3}}{10}\right)q. \end{array}$$

***Expressions for Q*_p1_–*Q*_p2_:**

$$Q_{p1}=\left(L_{1}{}^{3}L_{c}-L_{c}{}^{4}+\frac{5L_{1}{}^{3}L_{s}}{16}-\frac{5L_{c}{}^{3}L_{s}}{4}-\frac{3L_{c}{}^{2}L_{s}{}^{2}}{8}\right)p,\;Q_{p2}=\left(L_{1}{}^{2}L_{c}-L_{c}{}^{3}+\frac{5L_{1}{}^{2}L_{s}}{16}-\frac{15L_{c}{}^{2}L_{s}}{16}-\frac{3L_{c}L_{s}{}^{2}}{16}\right)p.$$

***Expressions for D*_1_–*D*_13_:**

$$\begin{array}{l} D_{1}=\frac{2}{5}\left(L_{c}{}^{5}-L_{x}{}^{5}\right)-L_{1}{}^{3}\left(L_{c}{}^{2}-L_{x}{}^{2}\right)-L_{1}{}^{3}L_{c}L_{s}+L_{c}{}^{4}L_{s}-\frac{L_{1}{}^{3}L_{s}{}^{2}}{5}+\frac{4L_{c}{}^{3}L_{s}{}^{2}}{5}+\frac{L_{c}{}^{2}L_{s}{}^{3}}{5},\;\\ D_{2}=\frac{1}{2}\left(L_{c}{}^{4}-L_{x}{}^{4}\right)-L_{1}{}^{2}\left(L_{c}{}^{2}-L_{x}{}^{2}\right)-L_{1}{}^{2}L_{c}L_{s}+L_{c}{}^{3}L_{s}-\frac{L_{1}{}^{2}L_{s}{}^{2}}{5}+\frac{3L_{c}{}^{2}L_{s}{}^{2}}{5}+\frac{L_{c}L_{s}{}^{3}}{10},\;\\ D_{3}=6h^{4}\left(\frac{1-\frac{8}{3}n^{2}+\frac{96}{5}n^{4}}{\frac{16}{3}n-\frac{128}{5}n^{3}}\right)\left(-\frac{2}{3}L_{m}+\frac{4L_{\mathrm{cs}}{}^{2}}{L_{m}}-\frac{8L_{\mathrm{cs}}{}^{3}}{3L_{m}{}^{2}}\right),\;\\ D_{4}=4h^{3}\left(\frac{1-\frac{8}{3}n^{2}+\frac{96}{5}n^{4}}{\frac{16}{3}n-\frac{128}{5}n^{3}}\right)\left(-\frac{2}{3}L_{m}+\frac{4L_{\mathrm{cs}}{}^{2}}{L_{m}}-\frac{8L_{\mathrm{cs}}{}^{3}}{3L_{m}{}^{2}}\right),\;\\ D_{5}=\frac{\left(1-4\alpha^{2})L_{m}{}^{2}(3+16n^{2}\right)}{24nE_{\mathrm{mc}}A_{\mathrm{mc}}}\left(\frac{1}{\frac{16}{3}n-\frac{128}{5}n^{3}}\right)\left(-\frac{2}{3}L_{m}+\frac{4L_{\mathrm{cs}}{}^{2}}{L_{m}}-\frac{8L_{\mathrm{cs}}{}^{3}}{3L_{m}{}^{2}}\right),\;\\ D_{6}=L_{1}{}^{3}L_{b}{}^{2}-\frac{2L_{b}{}^{5}}{5}-L_{1}{}^{3}L_{c}{}^{2}+\frac{2L_{c}{}^{5}}{5},\;D_{7}=-L_{1}{}^{3}L_{b}{}^{2}+\frac{2L_{b}{}^{5}}{5},D_{8}=L_{1}{}^{2}L_{b}{}^{2}-\frac{L_{b}{}^{4}}{2}-L_{1}{}^{2}L_{c}{}^{2}+\frac{L_{c}{}^{4}}{2},\;D_{9}=-L_{1}{}^{2}L_{b}{}^{2}+\frac{L_{b}{}^{4}}{2},\;\\ D_{10}=-2\cot^{2}\theta \left[2h^{2}(h_{c2}{}^{3}-h_{c1}{}^{3})-h(h_{c2}{}^{4}-h_{c1}{}^{4})+\frac{1}{5}(h_{c2}{}^{5}-h_{c1}{}^{5})\right],\;\\ D_{11}=2\cot^{2}\theta \left[2h^{2}(h_{c2}{}^{3}-h_{c1}{}^{3})-h(h_{c2}{}^{4}-h_{c1}{}^{4})+\frac{1}{5}(h_{c2}{}^{5}-h_{c1}{}^{5})\right],\;\\ D_{12}=-2\cot^{2}\theta \left[h(h_{c2}{}^{3}-h_{c1}{}^{3})-\frac{1}{4}(h_{c2}{}^{4}-h_{c1}{}^{4})\right],\;D_{13}=2\cot^{2}\theta \left[h(h_{c2}{}^{3}-h_{c1}{}^{3})-\frac{1}{4}(h_{c2}{}^{4}-h_{c1}{}^{4})\right]. \end{array}$$
